# Learning analytics: state of the art

**DOI:** 10.1007/s12008-022-00930-0

**Published:** 2022-06-18

**Authors:** Marcela Hernández-de-Menéndez, Ruben Morales-Menendez, Carlos A. Escobar, Ricardo A. Ramírez Mendoza

**Affiliations:** 1 Tecnológico de Monterrey, School of Engineering and Sciences, Ave. E. Garza Sada 2501, Monterrey, 64849 NL México; 2 General Motors, Global Research & Development, Warren, MI USA

**Keywords:** Educational innovation, Higher education, Learning analytics, Educational practices

## Abstract

*Learning Analytics* is a field that measures, analyses, and reports data about students and their contexts to understand/improve learning and the place in which it occurs. Educational institutions have different motivations to use *Learning Analytics*. Some want to improve students' outcomes or optimize their educational technology and reduce the dropout rate and others. This concept is presented with practical experiences that have been acquired and validated by 16 institutions. Besides, an analysis of the results, challenges, and expectations was performed. It was found that the majority of initiatives use *Learning Analytics* to improve retention of students; few are focused merely on improving the teaching/learning process or academic issues. The organizations invest their resources in acquiring *Learning Analytics* software; however, most universities develop their technology. The technology helps organizations be preventive and not reactive as various models determine students at risk of failing. This information allows them to make suitable interventions, which increases the success of the initiative. *CoViD19* pandemic is also put in context in this research; *Learning Analytics* could be a great approach to help the educational community adapt effectively to the new forms of educational delivery. Based on an exhaustive bibliographic review, various educational projects and experiences were analyzed, presenting an overview detailing applications, results, and potentialities and opportunities, hoping that this article will be a useful reference for researchers and faculty to exploit *Learning Analytics* education.

## Introduction

Higher Education Institutions (HEI) have different motivations for using Learning Analytics (LA). Some of them are aware of a student success movement that is arising as international studies have found that reasons for dropouts are related to the choice of the wrong program, have no motivation, personal situations, an unsatisfying first-year experience, absence of university support services, and academic unpreparedness (Ifenthaler, Yin, & Yau, [32]. Others want to get public funding, which is a tie to academic performance and want to improve the outcomes of the students or optimize their educational technology (Arroway, Morgan, O’Keefe, & Yanosky, [3]. Whatever the motivation is, LA is a field that has existed since 1979 when The Open University (UK) could analyze ten years of progress of their thousands of distance students [25]. Since then, and thanks to the widespread emergence of online learning and big data, LA has been in the spotlight of educational discussions. However, its adoption has been mainly done at the professor level, but it is a tool that is considered valuable to improve teaching and institutional management. The educational sector has had problems determining the value and impact of LA in enhancing learning. Their motivations for adopting LA include improving (1) teaching, (2) satisfaction and (3) retention of students, and (4) additional benefits for the institution and staff [69]. Table 1 summarizes the acronym definitions.

**Table 1:** Acronyms description

Acronym	Definition	Acronym	Definition
CSBA	Computer-Supported Behavioral Analytics	IT	Information Technology
CSLA	Computer-Supported Learning Analytics	ICT	Information and Communication Technology
CSPA	Computer-Supported Predictive Analytics	LA	Learning Analytics
CSVA	Computer-Supported Visualization Analytics	LMS	Learning Management Systems
EDM	Educational Data Mining	OER	Open Educational Resources
GPA	Grade Point Average	TEL	Technology-Enhanced Learning
HEI	Higher Education Institutions	VLE	Virtual Learning Environment

The use of digital tools in education generates many data and experiences from various sources such as online pedagogical platforms, academic enrollment, libraries, information systems, online assessment, social networks, etc. Any user's digital behavior can be tracked and analyzed to generate what have been called digital footprints. This significant amount of data can be recorded and diagnosed with many different technologies. LA provides educational leaders with the knowledge to improve the teaching/learning process [5], Boyer & Bonnin, n.d.). It is stated that online technologies could increase education quality [46]. They can also aid during air pollution crises, natural disasters, or pandemic diseases such as *CoViD19*, spreading rapidly worldwide and causing educational institutions to close their doors and thousands of people to be affected regarding their learning process. *UNESCO* states that it is necessary to "provide alternative modes of learning and education, and put in place equivalency and bridging programs, recognized and accredited by the state, to ensure flexible learning in both formal and non-formal settings, including in emergencies" [29]. Several challenges arise when using online learning in times of disruption [29]: (1) faculty/students feel alone, (2) demotivation of students, and (3) no time to adapt to change.

The effectiveness of the online digital learning process depends on the material prepared, the faculty's engagement in the new form of teaching, and the interaction that can be developed between faculty and students. From the side of the student, this learning demands more self-discipline (Aristovnik, Keržič, Ravšelj, Tomaževič, & Umek, [2].

A range of technologies compose online learning; it ranges from a simple tweet to an avatar-based simulation. Whatever the technology used, rushing to online learning could be a challenge. Some recommendations for doing this task are [62]:

- Manage students and faculty. They need to develop skills and behaviors, exploiting the current LMS.
- Divide lectures and mix them with different learning activities. Again, there are multiple platforms for doing this.
- Use online tutorials (these aid in acquiring knowledge and skills and are tools that engage students), videos, social media, MOOC, etc.
- Encourage online reflection by making students share their experiences with others and solve real-world problems.
- Demonstrate the value for active professionals of the shift to online learning.

LA can be used as a diagnosis and predictive tool. It can help determine student retention, performance, engagement, employability, progression, attainment, and mastery [5, 32], especially under critical situations such as the *CoViD19* pandemic. It can also be a tool for developing an open assessment by giving the learner timely feedback and explaining why such feedback was given. This process is essential as it could promote self-reflection and self-assessment competencies [17]. However, although LA has excellent HEI potential, it has not been widely used in this sector yet [5]. The lack of LA adoption in HEI can be explained by the absence of participation of teachers and communication issues between stakeholders such as professors, students, institutional directors, etc. However, research in the area has increased related to papers published. These investigations explore LA's use to increase retention, predictive analytics, social network analysis, discourse analytics, supporting students learning and determining its link with educational theory and learning design [34].

Due to the *CoViD19* pandemic, MOOC platforms have had an exponential expansion. e.g., Coursera grew 640% in the mid-March period to mid-April compared with the same period last year. This growth is not only the result of a provider's strategy of giving free access to over 3,800 courses; but as a need due to the pandemic spread [33]. MOOC use is the only alternative to give continuity to the teaching/learning process in some places. Various technological tools have been used during the *CoViD19* pandemic, including Zoom, Google Hangouts, Skype meets up, Google classrooms, and YouTube. Teachers need to rapidly adapt to the new technological environment, which poses a challenge [65]. Still, it is also a benefit as the crisis has made professors develop digital competencies quickly. However, no one knows what will happen after the crisis if professors continue to give classes in an online format or return to face-to-face ones. This will depend on their experience during the *CoViD19* pandemic [38].

The pandemic changed the educational dynamic, and its effect will be seen in the new normality in educational institutions. *CoViD19* will change how the future workforce is educated. Online teaching was mandatory during this phase of turbulence. Real-time video conferences, sending presentations to students, video recording, and written communication using forums and chats were the most formats used. These forms of education can be a challenge; learners need to be more conscious of their learning process to achieve educational goals. Teaching staff and universities' public relations were the entities that offered them significant support during the pandemic. Besides, students felt they were not performing well due to a lack of computer skills and a high workload. Also, they felt bored, anxious, and frustrated regarding their future professional career [2]. LA will be an essential tool in understanding and analyzing students' and faculty's digital footprints and experiences.

LA allows students to have information regarding their performance and what they need to do (practical guide and feedback) to reach their educational goals. They are conscious about how they learn, which is achieved by giving them continuous formative feedback. They can also compare with peers' performance; this adds a competitive element that is desirable for many students; because it develops an additional engagement. Learners can select future courses based on their past performance; this allows them to have an optimum and challenging pathway through their career choices. Adaptive learning systems are under construction to help students develop competencies and knowledge more personalized and adaptable. From the side of faculty, LA mainly permits them to know how effective is their performance/content and enable a continuous enhancement of it (Sclater, Peasgood, & Mullan, [63].

Faculty could track students' interactions with the system and make interventions opportunely, e.g., if a student has not read a post for a long time, the professor can intervene and determine what is happening. Also, suppose a student asks many questions regarding the material. In that case, faculty can access the learning environment and determine when and how often they access relevant tools of such a learning environment [20]. For the institution, LA allows knowing the general effectiveness of the learning programs. This is done by combining students' learning data and other educational data developed from the institution's different departments/offices [63]. Finally, LA will be a valuable tool for analyzing the new normality derived from the CoViD19 pandemic in an educational context. It will be a tool to determine the unique conditions faculty and students are teaching and learning.

LA has been developed by three main factors that are considered drivers of the field [25]:

1. *Online learning*: Students may feel alone due to the lack of contact with peers/teachers; they may have technical problems; they may lose motivation in virtual settings. This becomes particularly critical under the *CoViD19* conditions, where online teaching/learning/communication/etc. became a unique need and widespread; and.
2. *Political issues*: educational institutions are asked to demonstrate and improve performance. As well as for international recognition in academic rankings.
3.  *Big data*: there is a significant quantity of data obtained from the use of different educational software/platforms, with main characteristics such as high volume, velocity, variety, veracity, and value [17].

As mentioned before, LA leverages the digital footprint left by the virtual learning environments. These environments offer a significant quantity of information and data regarding user activities, which can improve the teaching/learning processes [59]. However, the data generated is complex, large, and heterogeneous, difficult to understand. Here is where LA techniques can help users understand the data and transform it into the information that can be used to make decisions (Vieira, Parsons, & Byrd, [71], especially in unexpected conditions, such as the massive move to online and/or virtual versions (with very different technologies) due to *CoViD19* pandemics. LA is one of the fastest-growing research areas related to education and technology. Using techniques such as predictive modeling, user profiling, adaptive learning, and social network analysis, LA uses data patterns to make recommendations for improving education [12], including new possible ways of teaching/learning.

Interest in LA is high among educational institutions; however, adoption remains low even though the HEI sector is incrementing its use to understand better and support students learning [70]. HEI is an education area that uses LA with different focuses: research, knowledge exchange, and praxis [45]. HEI research related to LA has been in [34]: student retention, predictive analytics, discourse analytics, helping students learn, and the relationship between educational theory and learning design.

LA has mainly been used to increase the retention of students and success and create personalized learning. It is stated that study success is affected by several factors, such as individual attitudes and characteristics of the educational environment. In a VLE, some factors contribute to determining students' success; these include predictors and visualization tools. Predictors are varied, e.g., forum interactions (posts, replies, etc.), engagement with learning options (videos, lectures, etc.), demographics, socioeconomic information, past academic experience and performance, and educational history. Visualization is done using dashboards [32].

Interest is growing in using LA to monitor the progress of students. However, LA is not a priority for universities. The research aimed at investigating the state of adoption of LA in Australian universities revealed that 2 out of 32 organizations that were studied reached the advanced stage of implementation. The other universities were in the preparatory or early stages of implementation. In the United Kingdom, results are similar; from 53 organizations surveyed, 25 did not implement LA, 18 worked on it, nine partially implemented it, and only one fully implemented LA [68]. However, publications and hence the interest in the area is growing,a study aimed at determining the adoption of LA in HEI found that 60% of publications were related to the theme of interest. On a global scale, the *USA* has been the leader in publications [30, 69], followed by Spain, the UK, Australia, Germany, Canada, India Netherlands, Japan, and China. As an example, the USA emphasizes its research in monitoring or measuring the students' progress. In summary, the adoption of LA in *HEI* globally is in an embryonic stage [69].

A study aimed at determining the four publication areas of LA identified and grouped related studies in categories (Charitopoulos, Rangoussi, & Koulouriotis, [15], Table 2.

**Table 2:** Publication areas of LA research

Area	Categories	Area	Categories
Results	Assessment and evaluation	Quality	Interaction between learner & platforms
	Prediction learning outcomes		Assessment of the system
	Decision making systems		Teaching quality assessment
Indicators	Student dropout rates	Support	Recommendation & guiding systems
	Learning style preferences		Recommendation systems for faculty
	Acceptance and enrollment rates		Structure of knowledge domain

**Fig. 1:**
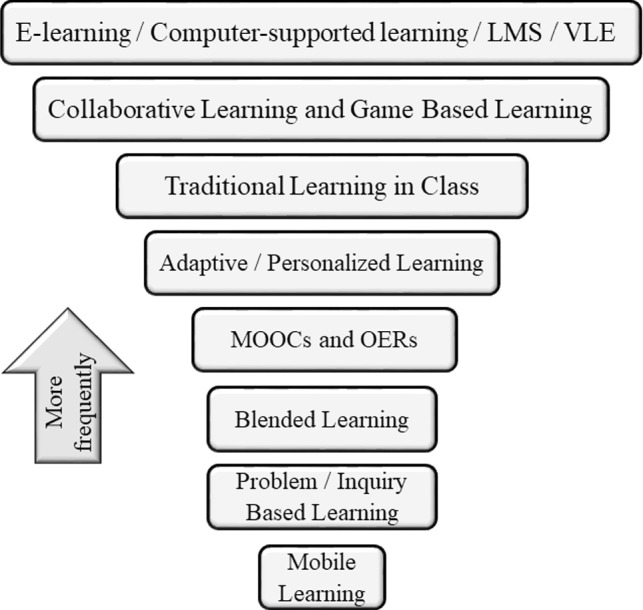
Learning context in LA research

It was also found that learning context used in LA research is varied, Fig. 1 [15].

Research in LA has a weakness, the lack of large-scale, longitudinal, and experimental studies related to its impact on learning/teaching in HEI. This is a big challenge for LA future research [32].

It is possible to determine if an organization is ready to adopt LA. *EDUCAUSE*  [3] has developed maturity indices to help institutions know where they stand. The LA maturity index measures 32 items, organized into six categories: (a) Decision-making culture including leadership agreement and acceptance of analytics, (b) Policies including data collection and access, (3) Data efficacy, availability of tools/software, (4) Investment & resources consisting of funding, (5) Technical infrastructure, store/manage/analyze data and (6) Institutional research involvement.

Each dimension is assessed with statements in which the interviewees express their degree of agreement. Each dimension's maturity score is the average of all responses, and the overall maturity score is the mean of each dimension score.

An intense literature review of LA was performed. The concept is presented with practical experiences that have been acquired and validated by different institutions. Besides, the results, challenges, and expectations were analyzed. The outline of this paper is as follows: Sect. 2 describes the concept of LA. Section 3 presents the experience that different organizations have had on the theme; many bibliographic references are included. Section 4 analyzes the results and generates some recommendations and guidance. Finally, Sect. 5 concludes the paper.

## Learning analytics

**Fig. 2:**
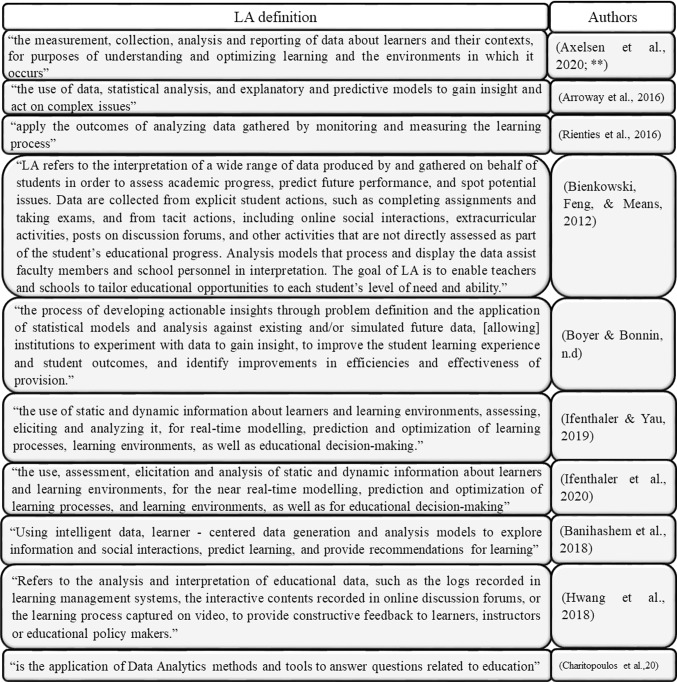
LA definitions (** Banihashem, Aliabadi, Ardakani, Delaver, & Ahmadabadi, [6, 7],Bellini, Santis, Sannicandro, & Minerva, 2019; [12, 16, 25, 28, 37, 49],Pazmiño-maji, García-peñalvo, & Conde-gonzález, 2016; [55], [60], [63], [71]

LA is one of the areas of Technology-Enhanced Learning (TEL) research that is growing fast. It consists of analyzing educational data to enhance the learning experience. For example, it can, e.g., determine the time a student spends on a specific activity and the number of visits to it. By combining the above data or any trace data with demographics and performance history, professors can personalize students' learning and redesign their courses, if necessary, according to a group of students (Rienties, Nguyen, Holmes, & Reedy, [57]. Some definitions of LA that have been proposed are deployed next, Fig. 2.

Regardless of the definitions, all of them know that learning data is obtained to improve the teaching–learning process. LA focuses on the relationship between the student and the learning environment. The final goal is to enhance students' success, defined as completing individual learning tasks and the successful obtainment of a degree [15, 31]. In the above definitions, three parts are noticed, Table 3 [53].

**Table 3:** Parts of LA definition

Input	Process	Output
Students learning data, demographics, perceptions and school processes	Integrated of four stages: measurement, collection, analysis and reporting	Understand and optimizing learning processes and environments

There are four forms of LA*:* descriptive analytics, diagnostic analytics, predictive analytics and prescriptive analytics (Boyer & Bonnin, n.d.), Table 4.

**Table 4:** Forms of LA definition

Form	Description
Descriptive (What happened?)	It examines the data and uses basic statistical techniques. E.g. it can be used to score the relative performance of the students
Diagnostic (Why happened?)	It uses techniques such as data discovery, pattern mining, or statistical correlations. E.g. identify if the teaching strategy is effective
Predictive (What will happen?)	It allows to anticipate the near future based on past events. E.g. determine which students are at risk
Prescriptive (How can we make it happen?)	It exploites tools able to process big graph analysis, text and data mining, simulation, etc

Data sources for LA are varied. The main one is the VLE, which accesses students to view course information, timetables, homework, etc. Another source is the information system of students in which data such as prior qualifications, grades, socioeconomic status, etc., can be encountered. Attendance monitoring systems and library data are also sources that can bring valuable information [63]. Data generated through the LMS and analyzed efficiently are [20]: (1) Number of times resource accessed, (2) date and time of access, (3) number of discussion posts generated and read, and (4) types of resource accessed.

There are studies in the literature in which data generated in the LMS has been used to predict students' performance. For example, researchers found that data such as login frequency, site engagement, student pace in the course, and assignment grades could predict course outcomes. Also, data such as the number of discussion messages read and the number of discussion replies posted can predict students' success [20].

The technical infrastructure is based on different technologies such as VLE, students information systems, business intelligence and visualization software, emerging LA packages, own house developments and enrollment, learning, and advising management tools [41], [63].

LA uses various techniques, including data visualization, artificial intelligence, data mining, machine learning, learning sciences, psychology, social network analysis, semantics, e-learning, and social aspects (Domínguez Figaredo, Reich, & Ruipérez-Valiente, [22, 55]. It also uses soft computing methods, including decision trees, random forests, artificial neural networks, fuzzy logic, support vector machines, and genetic/evolutionary algorithms [15].

**Fig. 3:**
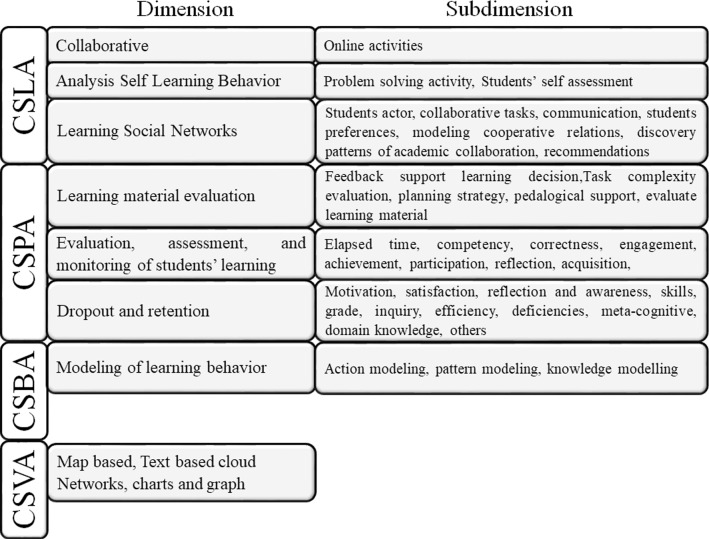
Dimension applications and techniques

Data mining is one of the techniques that is mainly used in LA. The methods can be classified into five groups: prediction, clustering, relationship mining, discovery with models, and separation of data for use in human judgment [4]. Authors [1] reviewed the literature and found a significant quantity of data mining techniques and their applications. They classify them in four dimensions:

- *Computer-Supported Learning Analytics* (*CSLA*) uses data mining algorithms to develop the interaction of students in the *LMS*. It identifies learning opportunities by assessing the exchange of students and their results.
- *Computer-Supported Predictive Analytics* (*CSPA*) is valid for predicting students' performance and retention by evaluating several dimensions, such as participation, engagement, and grades.
- *Computer-Supported Behavioral Analytics* (*CSBA*) shows students' behavior and preferences or motivations in a learning environment while participating in several different academic activities.
- *Computer-Supported Visualization Analytics* (*CSVA*) offers visual/graphical results related to individual behavior in a learning activity. Figure 3 presents different dimensions with their applications and techniques.

**Fig. 4:**
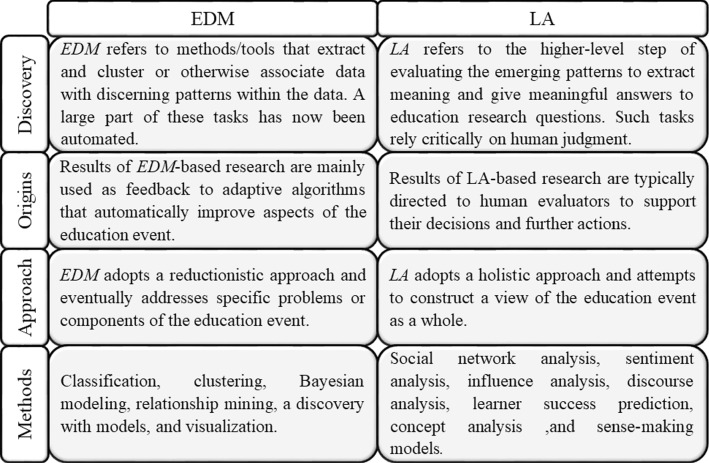
Differences between *EDM* and *LA*

There is a concept named *Educational Data Mining* (*EDM*), which uses data mining techniques for analyzing educational information; the difference between *EDM* and *LA* can be blurred. However, there are soft differences that are explained in Fig. 4 [15].

LA's other techniques are *Bayesian* modeling, natural language processing, and predictive modeling. Regardless of the method, all of them collect data about the learner and the learning process from various sources and improve and predict learners' success.

Some sources include the number of clicks, number of posts in a discussion forum, or the number of computer-assisted formative assessments attempted [56]. LA uses methods and processes for answering questions such as [9]:

1. What is the best time for a student to advance to the next topic in the course?
2. At what point is a student falling behind on a course topic?
3. When is a student at risk of failing a course?
4. What will be the evaluation of the performance of students without supporting them during the course
5. How should a student be administered based on their performance in a course?
6. How to determine the need for additional help managing a student

As can be seen, LA uses technology and the data generated through it to track students, if needed, help the students promptly, and be sure that learners will complete their careers. In addition, the LA process itself is preventive as it advances any situation that can cause a student to withdraw from his/her studies.

### Learning analytics process

There is no consensus about how the LA process should be; authors differ in the different stages it involves and the input data needed. [59] analyzed the strategies of other authors, Table 5.

**Table 5:** LA processes

Author	Stages
Author-1	1. What? (determine which data is going to be collected and analyzed)
	2. For Whom? (for which stakeholders is the analysis. Students, professors or directors)
	3. For what? (establish the purpose: monitoring, prediction, adaptation, personalization, etc.)
	4. How? (techniques, methods, and analysis tools)
Author-2	1. Sampling 3. Reduction
	2. Collection 4. Patterns finding
Author-3	1. Collection of “educational transactions” (big data)
	2. Information sources that can support the next stage: a) institutional system; b) big data; c) experience, intuitions, professors perspective
	3. Application of analytical software
	4. Decision making
Author-4	1. Collection of data 5. Analysis
	2. Storage 6. Representation and visualization
	3. Cleaning of data 7. Action (intervention, optimization, alerts, etc.)
	4. Integration 8. Restart the process
Author -5	1. Learning activities 3. Data storage and processing
	2. Data collection 4. Analysis
	5. Visualization 6. Action

As can be seen, several processes differ in various ways; some are very specific; others are too general. Some consider that an action must be part of the process (author-3, author-4, and author-5), while others do not. An interested person in implementing LA has to find its process. This procedure could be done by combining a different approach and refining it as the own process is running. Attention must be paid to the results; these must be measured to know the improvements made thanks to the intervention. It is also essential to have a leader who can negotiate the resources needed for the deployment [69], as LA is costly in terms of time, experience, and money [72].

### Learning analytics tools

Information Technology (IT) departments or LA specialists are the ones that design and implement LA tools [37]. Practical and well-designed LA tools reduce the time between the analysis and the action [17]. Some of the most known LA tools are listed in Table 6 [15], [25]:

**Table 6:** Some LA tools

LA Tools	Description
Social Network Analysis	It can investigate and promote collaborative connections between learners, teachers, and educational resources
GISMO	It is used for monitoring students taking into account the social, cognitive, and behavioral aspects. It presents graphical representations to analyze the above factor
CourseVis	It uses *LMS* data to help teachers know how their students perform in online classes and identify those who need extra support
Contextualised Attention Metadata	It integrates information from sources such as office tools, web browsers, multi-media players, and computer-mediated communication. The goal is the attention of users
LOCO Analyst	It gives feedback regarding the quality of the learning process
SMILI Open Learner Modelling Framework	It offers a method for describing, analyzing, and designing open learner models
Social Nets Adapting Pedagogical Practice	It analyzes interaction patterns of courses that help detect learner isolation, creativity, and community formation
Honeycomb	It visualizes large datasets, specifically networks, including millions of connections
Gephi	It performs advance analytics; it permits filtering, clustering, navigation, and manipulation of network data
Sense.us	It supports asynchronous collaboration; graphical annotation and view sharing can be performed
Signals	It uses large datasets to real-time predict students that are in danger of failure

[26] developed an inventory of LA tools that have been created by e-learning vendors, universities, or collaborative projects. They have different purposes, e.g., they can alert students struggling with their performance and give on-time support. Others make predictions success of students. Some tools adapt the content of a course to the learner's needs. Specifically, for HEI, some tools are worth mentioning, Table 7.

**Table 7:** Higher education institutions LA tools

Tool Name	Description
Degree Compass	Using information about the enrollment of students gives recommendations on which courses to take to complete the degree. It suggests which classes they are more likely to finish
Knewton	It is an adaptive learning platform for personalized education. This suggests lessons to students based on their performance/behavior. It provides information about their progress (immediate feedback). Faculty can see each learning status of the learners
Loop	It can be integrated with *Moodle* or *Blackboard*, and it is used to understand the behavior of students in the LMS. It has different components that aid professors to track the performance of students on time
Open Essayist	It offers automatic feedback regarding essays with suggestions to improve their writing. It analyzes the text and gives graphical feedback (prominent words, key sentences, highlights structure)
OU Analyse	It predicts students at risk of failure in their studies using ML. The tool reports the aggregated prediction value of several models for all students of a module and the reason that underlies its prediction
Student Success Plan	It provides information and analysis regarding the support services of students (counseling and coaching) to improve retention, academic performance, persistence, graduation rates, and completion time
Tribal’s Student Insights	It uses academic information, demographics, and assessment results to predict the general performance of students and determine those at risk of failure. Educators can use the information to provide students support and monitor modules concerning their predicted performance
X-Ray Analytics	It visualizes behaviors in their *LMS* at multiple levels: a course, numerous courses, and intuitional. It predicts those students who are at risk of failure. It is helpful to make timely interventions

### Learning analytics applications

**Fig. 5:**
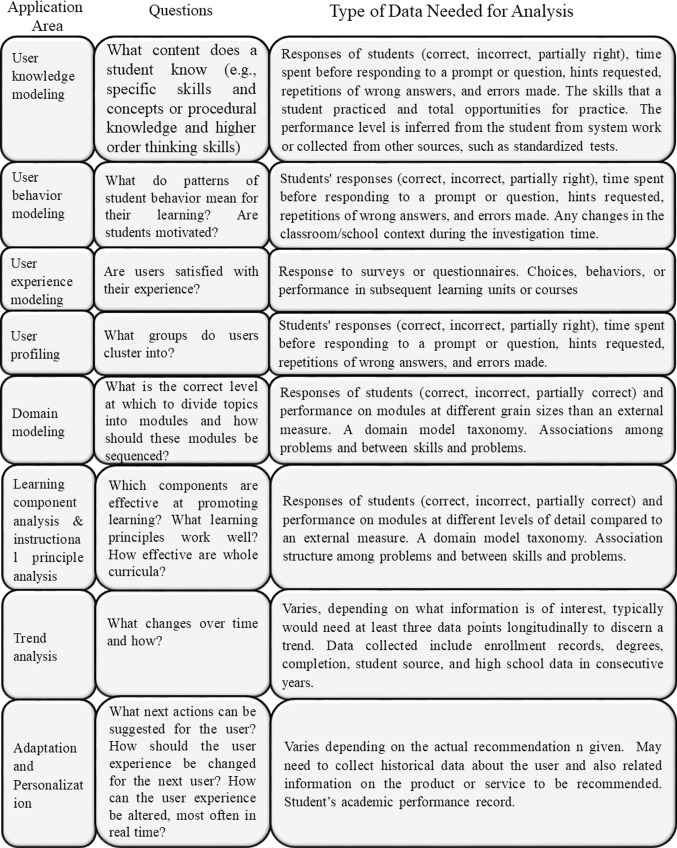
LA applications

LA applications are varied, these include the ones depicted in Fig. 5 [9].

Big data is one of the drivers of LA. Big data techniques have multiple applications in LA [14], [66]:

(a) *Performance prediction*. This can be done by evaluating students' interactions with faculty and peers in a virtual learning environment.

(b) *Risk detection*. By analyzing the students' behavior, the risk that students leave a course can be detected. Modifications can be done to the course bases on such analysis.

(c) *Data *visualization. Friendly visual reports can be developed thanks to various data visualization techniques that now exist.

(d) *Intelligent feedback*. Instant feedback can be offered to students based on their inputs. This feedback will improve the interactions of the students and their performance.

(e) *Course *recommendation. Courses can be recommended to the students based on their interests. This recommendation is made by analyzing their activities.

(f) *Student skills *estimation. Estimation of the skills acquired by the students.

(g) *Others*: grouping and collaboration of students, social network analysis, developing conceptual maps, constructing courseware and planning, scheduling, and identifying LMS users' behavior patterns.

### LA benefits and challenges

**Fig. 6:**
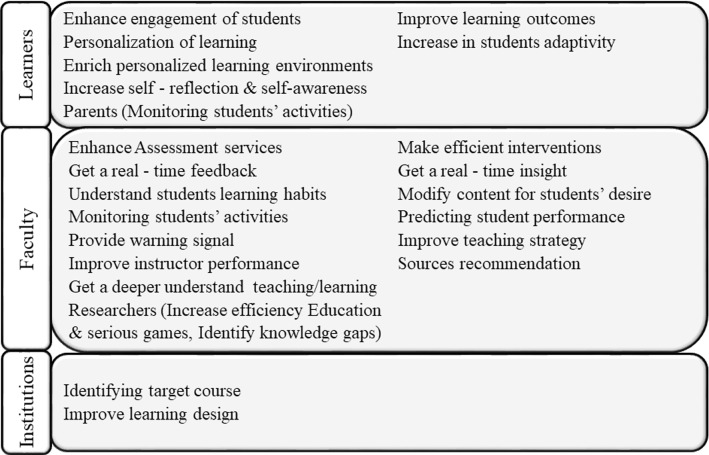
Benefits for different stakeholders

**Fig. 7:**
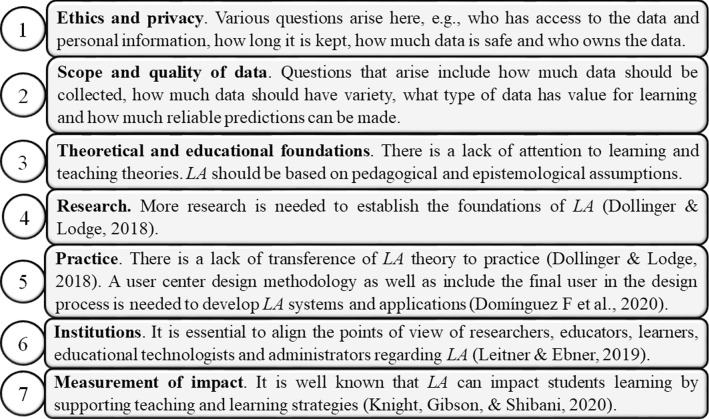
Challenges of LA

A group of researchers develops an investigation to determine the benefits and challenges of LA through a literature review, Table 8, and Figs. 6 and 7 [6].

**Table 8:** Benefits of LA

Benefits	Description
1	Increase the effectiveness of games when used in class
2	Increase engagement/results of learning in a *MOOC* collaborative environment
3	Help in understanding the learning behavior of students, modify content to align it to students learning characteristics
4	Predict the performance of the students and increase retention
5	Improve learning design and prevent that students leave their studies
6	Predict the performance of students and improve feedback
7	Useful to make evidence-based decisions
8	Improve curriculum, improve teacher performance
9	Understand how students learn
10	Identify knowledge gaps, improve teaching strategy

One of the benefits of LA is that it can offer personalization to the users. It is interesting to note that there is a model named the 70–20-10 that states that learning at the workplace is achieved through seminars, workshops, and eLearning courses (10%), followed by collaboration, mentoring, and coaching activities (20%) and personalized learning during the daily work (70%) [50].

## Experiences

A significant number of institutions are using LA in different ways and for various reasons. Some want to enhance students' experience by improving achievement, giving on-time feedback, and making students self-learners. Other organizations want to improve retention. *For example, Manchester Metropolitan University* increased nine percent satisfaction among the student's thanks to analyzing students' requirements, which indicated that the university should reorganize its curriculum. *Nottingham Trent University* uses LA to identify students at risk of failing and make interventions [63] opportunely. The experiences of organizations in LA are presented.

### Arizona State University (*ASU*)

*ASU* is committed to improving the students' success with the use of technology. In 2011, the university used Knewton Math Readiness software to improve the mathematics courses. This program developed a personalized learning path for 5,000 students enrolled in remedial mathematic courses [26]. Thanks to this strategy, the retention in such programs increases from 64 to 75%. *ASU* has also used *Civitas*, a data analytics platform, to improve students' success. This program helped the university to real-time track the performance of the students. It collects data related to class attendance, course participation, and the use of academic resources and support. *ASU* has been recognized for leading the student success movement by incorporating tracking systems, adaptive learning tools, big data, and predictive analytics. Due to this strategy, the *ASU*'s retention rate for 2018 was 85.2% (11% higher than 15 years ago). Also, the six-year graduation rate for cohort 2011 was 62.2% (6% higher than the national average of 57%) [54].

### Georgia State University (*GSU*)

*GSU* has used predictive analytics to improve the retention of students and graduation rates. *GSU* implemented the *Graduation and Progression Success* system to monitor the students' performance daily. As a result, the system helped the university have 1,700 more degrees in 2015–2016 than in 2011–2012. As a result of its strategy for improving students' success, *GSU's* 2017 retention rate was 83%, and its six-year graduation rate increased from 32 in 2003 to 53.7% in 2017 [54]. In addition, the university serves minorities,in research performed using predictive analytics, the institution found that students with extraordinary academic results dropped before graduation due to non-payment. Thanks to these highlights, graduation rates went from 32% in 2003 to 54% in 2014 [26].

### Rio Salado College

This college began to use data mining and predictive modeling research in 2008. In 2010, the institution developed the *RioPACE* model to determine students at risk. The idea was to have an alert system that identifies those students who are struggling academically. The program uses the naïve *Bayes* model to determine appropriate warning levels weekly using updated activity and grade information [58]. The system analyses the performance of current students and compares it with previously successful ones. It uses a color code that expresses the level of achievement weekly. The final goal is to make interventions opportunely for improving the students' success. Students also have access to the system. If the indicator is yellow or red, learners must contact their teacher for help [26].

### The Open University (OU)

OU has collected and analyzed data over many years. The knowledge generated has helped the organization support students promptly and retain them. The effort began in 2013 with a project to explore LA, focused on developing different analytic solutions (R. [26]. This effort's results are varied, e.g., comparing 40 learning designs at the OU and learner behavior, satisfaction, and academic performance. The way teachers design a learning module influences students' engagement over time [56]. OU has a policy document in which the treatment of the data generated through their systems is specified regarding privacy issues. It states that the data will be used only to improve the students' success. This policy is aligned with its principle of "treat each other with dignity and respect" [26].

### University of Alabama (*UA*)

This organization works with analytics to retain its students. An analysis of enrolled first-year students from 1999, 2000, and 2001 was used to develop students' predictive models, specifically those at risk. Techniques such as logistic regression, decision trees, and neural networks were used to make predictions. The model is composed of eight variables: (1) *UA* cumulative *GPA*, (2) English course, (3) English course grade, (4) *Distance* from *UA* campus to home, (5) Race, (6) Math course grade, (7) Total earned hours and (8) Highest *ACT* score. The model can identify 150–200 first-year students who will not finish their studies each year. This information is shared with the advisors to make interventions opportunely. The model is continually updated to adapt to students' new characteristics (Campbell, DeBlois & Oblinger,  [13].

### Sinclair Community College

This institution is committed to improving students' completion rates. Since 2000, approximately 100 completion-related projects have been developed. One strategy is to use *Civitas Learning*, a predictive analytic tool [24]. The organization also created the *Student Success Plan* (*SSP*) software to improve retention, graduation rates, performance, persistence, and completion time. It has been used for ten years and has gained 11 awards in the *United States*  [26]. The data is collected and analyzed quarterly for trends. It has improved students' learning outcomes, mainly for low-income and academically unprepared students who have problems with courses [48]. From 2005 to 2011, students that used *SSP* were five times more likely to graduate [26].

### Northern Arizona University (NAU)

This organization uses various resources to help at-risk first-year students. The university developed a model that comprises three main elements (critical in the process):

1. Resources/services utilization (academic services, recreational resources, social resources, academic referrals, advising/career sessions).
2. Level of risk (admissions test scores, high school *GPA*, and psychosocial factors).
3. Outcomes (first-year student *GPA* and enrollment retention status).

Results are promising, e.g., *the GPA* of students who used 1 to 3 academic services increased by 0.192. Students who used four services increased *their GPA* by 0.280 points. Finally, students at risk and using four services increased *their GPA* by 0.460 points. The variables that had the most significant impact on the model were academic referrals and advising/career sessions [13].

### Purdue University

This university uses its course management system to predict which students have problems with their studies and make interventions opportunely. This initiative's rationale is that students' academic success depends on students aptitude (e.g., test scores) and effort [13]. Based on the importance of LA for the university, the organization created Course Signals. This Course Signals is a predictive LA system that determines students at risk of not finishing a course. Making the analysis, students are assigned to a group coded by color (red, yellow, green). Data used consists of general information about the students and activities in the learning management system. Finally, the system sends emails to those students at risk,the university increases its retention by 21% [26].

### New York Institute of Technology (NYIT)

*NYIT* developed an in-house predictive model to identify students at risk. The goal was to increase first-year students' retention by identifying those who need support and giving specific information about each student to make interventions opportunely. The process consisted of mining the data, running the analytics, and developing friendly outputs that helped the counseling staff. Four data sources were used: admission application information, placement test data, a survey completed by all students, and financial information. The model had 74% recall and 55% precision. Results were presented in a table; one row was assigned to each student, indicating if he was probable to return the following year to classes; the percentage confidence of the prediction and reason for the prediction was added. With this information, tutors or counselors can talk with each student about the situation and propose plans [63].

#### University of Maryland, Baltimore County

This organization integrated into its *VLE* the *Check my Activity (CmA)* software. This tool helps students compare their activity in the *VLE* with a summary of the whole cohort’s activity. In a generation of students, 92% used the *VLE*, and of those, 91.5% used the *CmA* software. These data results are impressive as those who used the tool were 1.92% more likely to get a grade C o higher than those who did not. Research regarding the *VLE* found that one professor with high participation rates used *Blackboard*’s adaptive release feature to allow students to take quizzes before accessing the assignments. It was found that these students scored 20% higher than students in other sections. They also perform better in the following courses. In summary, analytics found that “effective implementation of a *VLE* tool on a prerequisite course may lead to enhanced performance not just in that course but also in subsequent courses” [63].

#### University of Wollongong

This organization developed the Social Networks Adapting Pedagogical Practice (SNAPP) initiative. The tool analyses students' conversations in online forums to find patterns in real-time through social network diagrams. They found that collaborative learning can aid in promoting students' understanding. Besides, the quality of professors' intervention in those forums significantly impacts students' learning experience. The tool can help instructors analyze how the group behaves over time and found students who are isolated or lead discussions. As the tool gives real-time information, professors can act on time to change, e.g., the learning strategy. They also can use it to make a general reflection after the course has finished. The SNAPP initiative revealed a strong correlation between students' learning interests and the forums they participate in [63].

#### Open Universities Australia (OUA)

*OUA* is an online educational group integrated by seven Australian universities. It uses the *Personalised Adaptive Study Success* (*PASS*) to plan the students’ curriculum personalized. The main goal is to support those students who have problems in their studies by suggesting alternative courses according to their specific needs. For example, a student with trouble in one topic can take extra modules to strengthen that area. The sources of information for *PASS* are varied, ranging from customer relationship management systems to the curriculum profiles for each unit and program. *PASS* uses *LA* to give students recommendations in real-time through a dashboard engine that can be customized [63].

#### Tecnológico de Monterrey (Tec)

*Tecnológico de Monterrey* has developed various academic administration procedures, exploiting LA and classifying students through policies in the academic regulations in three primary states: (1) regular student, (2) prevention student, and (3) conditioned student. Through data mining using educational information (i.e., current and past grades), behavior (i.e., class attendance), trends (current and rolling grade point average), and experience from the *Office of Student Academic Improvement*, the condition of the student who is enrolled (by regulation) in a specialized academic improvement program to help him improve his academic performance. This program has been continually enhanced and achieved excellent results with more than 15,000 students on campus. The main results are a reduction in the drop-out rate (~ 15%), a drop in the percentage of students who change careers (~ 20%), a reduction in the average study time of students (~ 15%), and a solution of the fundamental problems of students (~ 30%), etc. To date, most decisions are made through manual processes by highly qualified personnel (mainly with the profile of psychologists.)

#### University of Michigan – E.^2^Coach

In 2012 the *University of Michigan* began to use in the introductory physics class *E2Coach*, an *LA* system, to give students personalized feedback. The goal was to determine students’ success by predicting final grades. The system provides written feedback in personalized messages that include advice to prepare for an exam, how to use the system better, and feedback regarding student performance. Results indicate that the systems work well as users have an average of 0.11 and 0.18 points higher in their final grade, considering that non-users showed non-difference [64].

#### Dublin City University—PredictED

*Dublin City University* developed the project *PredictED* to improve the learning experience and students' performance in their final examinations. The software is running in 17 different first-year courses. The information sources are data from the *VLE* and past exam grades; by combining them, the university can determine if a student will pass or fail the course. The software also informs students how they perform; a weekly alert is sent to them, indicating if they need to study more. Results suggest that participants' average scores are significantly higher with the intervention. These results were determined by analyzing ten courses and a sample size of 1,270 students [23].

#### University in Ankara, Turkey

Researchers of this university developed an investigation to identify students’ behavior patterns in an online learning environment. Moodle platform was the tool used for performing the analysis. One hundred sixty-five students registered in the course Information and Communication Technologies. Data such as students’ logs and interactions were used, and results indicate that the technological tool elements that students mainly deal with are course modules and discussion forums. This information is valuable for teachers as they can trace students’ behavior and, if necessary, change the teaching items and strategy in the software [39].

In Table 9, the main elements of the initiatives previously described are presented.

**Table 9:** Main elements of LA initiatives

	Arizona State University	Georgia State University	Rio Salado College	The Open University	University of Alabama	Sinclair Community College	Northern Arizona University	Purdue University
*a*								
Goal	Increase retention	Increase retention and graduation rates	Increase retention and graduation rates	Increase retention	Increases retention	Improve completion rates	Increases retention	Increases retention
Application / Technology	Knewton Math Readiness /Civitas Learning	Graduation and Progression Success system	RioPACE model	Different analytic solutions	Predictive models (logistic regression, decision trees and neural networks)	Civitas Learning/ Student Success Plan	Own model	Course Signals
Result	Retention increases from 64 to 75% / in 2018 retention was 85.2%	1,700 more degrees in 2015–2016 than in 2011–2012. In 2017 retention rate was 83%	The model identifies the level of achievement of each student on a weekly basis and opportunely interventions are made	It was found that he way teachers design a learning module influences students engagement	The model is able to identify 150–200 freshmen each year who are not going to finish their studies	Have proven to be successful in improving students learning outcomes	GPAs of students increased between 0.192 and 0.460	Retention increased by 21%

	New York Institute of Technology	University of Maryland, Baltimore County	University of Wollongong	Open Universities Australia	Tecnológico de Monterrey	University of Michigan	Dublin City University	University in Ankara, Turkey
*b*								
Goal	Increases retention	Improve performance	Promote students understanding	Personalize students curriculum	Increases retention	Determine students success	Improve learning experience and performance	Analyze students behavior
Application / Technology	Own predictive model	Check my Activity	Social Networks Adapting Pedagogical Practice	Personalized Adaptive Study Success	Own predictive model (100% manual)	E^2^Coach	PreditED	Moodle
Result	The model had 74% of recall and 55% of precision	Those who used the tool were 1.92% more likely to get a grade C o higher	Professors are able to change teaching strategy on real time. There is a strong correlation between students’ learning interests and the types of forums that they participate on	The system give recommendation to students in real time through a dashboard engine that can be customized	Improve all performance indicator more than 15%	Users have an average of 0.11 and 0.18 points higher in their final grade	Average scores of participants are significant higher with the intervention	Students dealt mostly with searching and viewing of course modules and discussion forums

As can be seen, there is high interest from universities to invest resources in implementing LA projects. For all the universities, results from the LA process seem promising. The majority of institutions use LA to increase retention; this is their primary goal. Regarding the technology used, only a few use commercial software; the rest have developed their technological solutions or models.

## Results

Most initiatives previously presented use LA to improve students; few are focused merely on improving the teaching/learning process or academic issues. The reason could be desertion rates are high because students are not engaged, are not prepared to finish their studies, have financial problems, or are isolated socially talking. Various strategies have been proposed. One option is to personalize learning with the use of technology. The organizations invest their resources in acquiring LA software. However, the majority of universities develop their technology. Civitas is software that some universities have used with good results. Others, such as Georgia State University, Rio Salado College, University of Alabama, Northern Arizona University, Purdue University, and New York Institute of Technology, have developed their models.

The technology helps organizations be preventive and not reactive as various models determine students at risk of failing. This advice allows them to make reasonable interventions, which increases the initiative's success. In addition, pour information was found regarding privacy issues. OU is the only found university that specifies how the data collected will be treated.

The results have been good. In those cases where information was available, retention rates vary from 11 to 21%. Others have retention rates above 80%. Additional results indicate that LA can predict performance results, used to make suitable interventions. Some universities have found cause/effect results, e.g., Purdue University found that academic success depends on student aptitude and effort. In the case of OU, it was found that the way teachers design a learning module influences the students' engagement.

Those who want to implement LA in their institution need first to see and analyze what others have done, which is the goal of this work. The majority of institutions included have been experimenting with LA for years. This process takes time, which is "personal" for each institution. Also, benefits and challenges must be considered to decide how and where to implement LA. The effectiveness of LA has to be studied to deepen. More results are needed to determine that LA improves the teaching–learning process. This research needs to be done for specific contexts and problems. The benefits of LA in education are numerous:

1. Identify courses that are more likely to fit with students' interests and preferences;
2. Obtain data that will allow for improvement or change in the curriculum;
3. Determine student's actual outcomes and improve their performance;
4. It is possible to personalize the learning of each individual;
5. Improve the performance of professors as the institution can analyze their technological behavior;
6. The use of big data allows to identify of post-education employment opportunities and permits to align of education with market needs;
7. Analysis can be made so that researchers encounter gaps between industry needs and academia.
8. The most significant benefit of LA is that it permits to perform early interventions when a student is facing difficulties (Kollom et al. 2020)

Some challenges must overcome.

1. Data tracking, which is helpful to see individuals' performance, depends mainly on the platform used (Moodle, Canvas, EPIC, Blackboard);
2. Data collection can be a challenge, and it has to be delivered in a timely and accurate manner, which is not possible with today's systems;
3. Technical challenges also exist in the analysis of information. There is a need for technical resources to manage big data;
4. Researchers must discover insights from the users' perspective of the learning systems. Then, more advanced data sets such as mobile, biometric, and mood data are needed;
5. To take advantage of the full potential of LA, a technology that is still under development is required;
6. Ethical and privacy issues also need to be considered. Today privacy and control of information affect the adoption and deployment of LA systems [4],
7. There is a need for systems that give real-time feedback to the users, i.e., systems that offer information as the learning activity is ongoing [10],
8. Researchers are still working on reducing computer capacity to store big data. However, experts are not so optimistic regarding this issue [9]
9. There is a lack of large-scale studies regarding LA and its impacts on learning and teaching in HEI [32].
10. LA systems have been developed without the active participation of students and teachers. They only have an observational role. The design and implementation of LA tools are mainly the responsibility of the IT department and learning analytics specialists.
11. It is not clear if LA offers a positive effect on learning. This is because educational institutions are more interested in grades, persistence, and non-completion metrics than students' motivation, engagement, satisfaction, and more formative learning assessment [27].
12. Data accuracy and understandability are the most critical challenges that professors consider must be faced (Kollom et al. 2020)
13. Many companies do not know what to do with all their generated data [44].,this is the case for education and manufacturing organizations.

It is stated that LA requires many resources in money, time, and experience [37]. Also, a question arises if digital traces as proxies for learning are helpful and measure learners' performance effectively [49]. On the other hand, studies indicate that LA has successfully allowed students to complete their courses or continue with them [32]. LA has also been used to evaluate if students acquire lifelong learning competencies. Skills such as problem-solving, logic, debugging, creative thinking, analytical thinking, conceptual thinking, self-efficacy, and time management are skills assessed with LA's aid [36].

Educational technology has been commercialized, and authors report that some platforms use "psychological, behavioral management techniques and rankings to model student behavior according to the system." This falls in the area of ethics and opens the question of whether these platforms improve learning. There is also a concept arising that has to do with LA.

The idea is the datafication of education based on business intelligence. It is stated that all the data generated is mainly used to influence people's behavior. This is the other side of LA (Teräs, Suoranta, Teräs, & Cur, [67]. However, the narrative of LA promotes student engagement and personalized learning [19] by processing a large amount of data that needs to be used to improve education. The rationale behind this is that the more data generated, the better. There has to be an unrestricted flow of information if this goal wants to be achieved [18]. Numerous sources of information are available for making LA. LMS are ideal as they offer a fertile ground of information. Also, massive open online courses (MOOCs) are another data source. These data can be combined with other information such as socio-demographics, course engagement data, entrance grades, test results, and library usage [27]. LA can also be combined with other disciplines such as psychology, educational science, and computer science [42],with this, the sources of information can be infinite. Other disciplines in which LA has been applied are decision science, social sciences, engineering, mathematics, arts and humanities, nursing, business management, and accounting and medicine [47]. Regarding LA applications, the research performed by [61] offers a significant number of useful tools.

Regarding privacy issues, institutions must consider that a large amount of information generated also poses a responsibility regarding how this information will be used. Researchers think that students have the right to limit data analysis practices and express their privacy preferences to control their information. This issue has to be considered as it could cause problems in the future development of LA [35]. There is a need to determine who has access to which data, where and how much data will be deposited, and which algorithms and procedures should be implemented. Aspects that need to be considered include who has access to which information, where the data will be stored, and how long and which algorithms will be implemented [32]. Authors argue that research has demonstrated that students are unaware of the use of their data for LA. They do not even know which of their data is collected [43].

Set a clear strategy is needed for implementing LA in an educational institution. Also, investment in infrastructure has to be considered, and ethical and privacy considerations have to be set from the beginning. From the side of the educators, they need support to have systems that give them immediate results. A professor cannot take timely actions with data generated various months ago. They need on-time information to help students of tomorrow [9]. One exciting thing about LA is that it can infer trends across educational organizations, programs, classes, etc. It provides feedback to individual students and professors [40]. However, the efficacy of LA depends on the skills that professors have in making interpretations of the data and thus providing actionable feedback [42].

Some recommendations for implementing LA specifically at the HEI country level include:

1. Establish data standards,
2. Identify the requirements for data collection,
3. Introduce privacy, ethics, and data protection standards.
4. Promote the efficient use of data standards,
5. Make sure data is associated with metadata with formal/standard procedures
6. Have benchmarks to compare yourself
7. Collaboration with professionals,
8. Exchange experiences
9. Integrate into university networks and link with society
10. Promote knowledge exchange, especially between disciplines.
11. Support to institutions,
12. Promote linkage models between institutions for their adoption and transfer.
13. Support institutions in evaluating available resources.
14. Establish diligence mechanisms to develop interventions,
15. Give autonomy to universities in the administration of their data

At the school level, some recommendations include:

1. It is essential to have democratic control,
2. Data use considerations,
3. Need for capacity building,
4. Focus on ethical questions [31].

The future is narrow for LA as it is still a new field of study; i.e., it is in an early development stage, so it isn't easy to make predictions. However, researchers have developed some bets by looking at the experience of other industries. Gartner has predicted that LA will become more automatic and that data from all sources will be used. Also, privacy and ethical aspects will have more attention. On the other hand, access to the core algorithms in predictive systems will be possible. Besides, more advanced and personalized dashboards will be developed for students and professors. These will make an advanced analysis of raw data and the content of the work and a score of competencies [3]. LA in education will provide shortly personalized and rich learning on a large scale [56]. Experts in HEI believe that soon LA will be significantly used in online education to identify student behavior patterns and improve learning and retention rates [6]. However, even though the importance of LA, there is missing a widespread application of it in HEI. Indeed, LA has a significant impact on those organizations, and many have not yet exploited the data generated to address the challenges they face [5]. Other authors consider that even though LA is a new area, it has matured enough in HE application [40]. Also, publications on LA have grown at a fast pace since 2011. The growth is in terms of the techniques, methods, and applications offered [47]. Developed countries, such as the USA and those from Europe, are the ones that lead research in LA [52].

LA will also influence the development of the Industry 4.0 approach. The *Internet of Things* and *the Internet of Educational Things* will significantly impact the studies of *LA*  [60]. Multimodal LA is another term that is emerging. It integrates data from different sources, such as physiological and contextual data. Various researches in this regard have been developed. One interesting is the one in which a tool called Lelikëlen and a Microsoft Azure Kinect camera were used to capture students' postures while presenting. Results indicate that there were statistical differences in students' behavior while offering, and this, combined with teachers' additional information, can give interesting results (Morán-Mirabal, [51]. Multimodal LA is more robust regarding the understanding of the learning process. It incorporates sensor data that capture gestures, gaze, or speech. Therefore, it gives a complete outlook of the learning process [34].

## Conclusions

LA is an emerging field taking the attention of the learning community as it offers valuable tools and processes for improving the teaching–learning process. The definition of LA most adopted by authors is "the measurement, collection, analysis, and reporting of data about learners and their contexts, for understanding and optimizing learning and the environments in which it occurs." It is composed of input (data), a process (analysis), and an output (optimize learning). Whatever the definition used, LA has proven to enhance the success and retention of the students.

Educational institutions that use LA are aware of the payoff in learning improvement and student retention that this approach offers. Professors can track students' progress and be mindful of the educational strategies that are most likely to work and be effective. Students can also analyze their progress, determine their strengths and weaknesses, and be promoters of their learning. Educational organizations are using LA to improve students' satisfaction and retention with good results. More research is needed to exploit at full its potential, mainly to determine the actual effectiveness it offers; on the other hand, there is a lack of investigation regarding students' points of view. It will be interesting to know what opinion they have regarding an issue of their real concern. The main challenges depicted in this work need to be taken into account, mainly privacy and ethical concerns, as these could severely impact LA development. Also, research needs to be pulled to practice as there is a lack of such transference.

Nowadays, it is necessary to personalize learning environments and let learners be creators of their education. LA is a practical approach that can explore how learners learn and support them in adjusting the learning environment to their needs. The final goal is to give them control of their learning process.

Research in LA has had great attention from the educational community. However, its application in real LA projects is still lacking. There is a need to transfer research into practice by applying a user-centric approach and including the final user in any course [21, 22]. In general, education research is incomplete, imprecise, and qualitative. There is an excellent opportunity to use educational data with *Artificial Intelligence*, and *Soft Computing* approaches. However, the results will depend on the quality of the input data [15].

Online technologies can aid in turbulent times, such as a *CoVid 19* pandemic, which has caused a redesign of the teaching–learning process and an option to give continuity to academic issues. This has posed a challenge and a benefit, allowing professors and students to develop digital competency quickly. In addition, all the data generated during this disruption can be analyzed with LA techniques and make education adapt to the new delivery forms effectively and to the new normality.

A review of LA was performed, where basic concepts were discussed. The practices that 16 educational organizations such as *Arizona State University, Georgia State University, Rio Salado College, The Open University, and the University of Alabama* have adopted regarding the theme are described. The majority of them use LA to retain students; others focus on improving academic issues. They have also developed in-house software or models. According to the organizations, results are promising as they have achieved good percentages of retention/improvements over the years.
